# Potential Cardioprotective Effects and Lipid Mediator Differences in Long-Chain Omega-3 Polyunsaturated Fatty Acid Supplemented Mice Given Chemotherapy

**DOI:** 10.3390/metabo12090782

**Published:** 2022-08-24

**Authors:** Austin Angelotti, Deena B. Snoke, Kate Ormiston, Rachel M. Cole, Kamil Borkowski, John W. Newman, Tonya S. Orchard, Martha A. Belury

**Affiliations:** 1 Department of Human Sciences, The Ohio State University, Columbus, OH 43210, USA; 2 Department of Medicine, University of Vermont Larner College of Medicine, Burlington, VT 05405, USA; 3 Division of Medical Oncology, The Ohio State University Comprehensive Cancer Center, Columbus, OH 43210, USA; 4 West Coast Metabolomics Center, Genome Center, University of California Davis, Davis, CA 95616, USA; 5 Western Human Nutrition Research Center, United States Department of Agriculture-Agriculture Research Service, Davis, CA 95616, USA; 6 Department of Nutrition, University of California-Davis, Davis, CA 95616, USA

**Keywords:** heart, omega-3 fatty acids, oxylipins, endocannabinoids, lipid mediators, chemotherapy, diet, mitochondria

## Abstract

Many commonly used chemotherapies induce mitochondrial dysfunction in cardiac muscle, which leads to cardiotoxicity and heart failure later in life. Dietary long-chain omega-3 polyunsaturated fatty acids (LC n-3 PUFA) have demonstrated cardioprotective function in non-chemotherapy models of heart failure, potentially through the formation of LC n-3 PUFA-derived bioactive lipid metabolites. However, it is unknown whether dietary supplementation with LC n-3 PUFA can protect against chemotherapy-induced cardiotoxicity. To test this, 36 female ovariectomized C57BL/6J mice were randomized in a two-by-two factorial design to either a low (0 g/kg EPA + DHA) or high (12.2 g/kg EPA + DHA) LC n-3 PUFA diet, and received either two vehicle or two chemotherapy (9 mg/kg anthracycline + 90 mg/kg cyclophosphamide) tail vein injections separated by two weeks. Body weight and food intake were measured as well as heart gene expression and fatty acid composition. Heart mitochondria were isolated using differential centrifugation. Mitochondrial isolate oxylipin and N-acylethanolamide levels were measured by mass spectrometry after alkaline hydrolysis. LC n-3 PUFA supplementation attenuated some chemotherapy-induced differences (*Myh7*, *Col3a1*) in heart gene expression, and significantly altered various lipid species in cardiac mitochondrial preparations including several epoxy fatty acids [17(18)-EpETE] and N-acylethanolamines (arachidonoylethanolamine, AEA), suggesting a possible functional link between heart lipids and cardiotoxicity.

## 1. Introduction

Antineoplastic drugs, commonly referred to as chemotherapy (chemo), have successfully increased cancer survivorship. However, as cancer survivorship increases, long-term adverse events resulting from chemotherapies have become prevalent [1]. A common side effect of chemo, especially regimes with anthracyclines, is heart damage (i.e., cardiotoxicity), which can lead to left ventricle dysfunction and congestive heart failure [2,3]. Anthracyclines cause cardiotoxicity through various molecular mechanisms including topoisomerase inhibition, which leads to DNA damage [4,5] and incorporation into heart mitochondria, leading to reactive oxygen species formation and mitochondrial dysfunction [6,7,8,9,10,11]. Through these known mechanisms, anthracyclines activate cardiomyocyte apoptosis and fibrosis, ultimately leading to cardiac hypertrophy and heart failure [12,13].

Dietary fish oil, containing the long-chain omega-3 polyunsaturated fatty acids (LC n-3 PUFA), eicosapentaenoic acid (EPA), and docosahexaenoic acid (DHA) have been widely studied for their cardioprotective properties [14,15,16,17,18,19]. The premise of these studies is generally based on EPA and DHA being incorporated into the cell membranes and altering the downstream lipid mediators involved in cellular communication [20].

Lipid mediators are bioactive lipids produced from both enzymatic (cyclooxygenases [COX], lipoxygenases [LOX], cytochrome P450 [CYP450], etc.), and non-enzymatic (auto-oxidation) pathways in response to external stimuli [21]. Many lipid mediators are formed through the oxygenation of the double-bonds present within fatty acids (termed oxylipins), while others (such as certain endocannabinoids, e.g., anandamide and 2-arachodonly glycerol) are formed by fatty acid amide or ester linkages to head groups such as ethanolamine or glycerol.

Lipid mediators regulate inflammation, immunity, fibrosis, vasculature, reactive oxygen species accumulation, and mitochondrial function [21,22,23,24]. Additionally, cardiovascular pathologies such as hypertension, ischemia, and heart failure have been linked to aberrant lipid mediator signaling [22]. Mitochondria present a unique compartment within cells due to their highly oxidative environment and ability to both generate [25,26] and catabolize oxylipins [27]. Notably, both auto-oxidative and CYP450-derived lipid mediators have cardioprotective properties preserving mitochondrial function [28,29]. For example, F4-neuroprostane, a non-enzymatic oxidation product of DHA, reduces heart damage from ischemia/reperfusion injury by increasing mitochondrial membrane potential and reducing apoptotic signaling [29]. CYP450-derived epoxy fatty acids (epoxides), a well-studied subclass of oxylipins, are cardioprotective in models of cardiovascular disease through anti-inflammatory, anti-fibrotic, and mitochondrial-preserving effects [30,31,32,33,34]. Variants in genes controlling epoxide synthesis (CYP450 enzymes) and metabolism (e.g., soluble epoxide hydrolase, sEH) are associated with cardiovascular disease risk [35,36]. Ultimately, lipid mediators are important regulators of cardiovascular health and mitochondrial function, and therapeutic modulation of their synthesis, degradation, or signal transduction may provide novel approaches to treat cardiovascular diseases.

Dietary LC n-3 PUFA interventions can alter membrane fatty acid and lipid mediator composition and increase levels of putatively cardioprotective metabolites in certain contexts [37,38,39,40]. This study aimed to test whether LC n-3 PUFA supplementation can attenuate chemo-induced markers of cardiotoxicity and mitochondrial damage. Due to chemo’s ability to induce lipid peroxidation and enhance the formation of reactive oxygen species within heart mitochondria [10], we also characterized the cardiac lipid differences between diet and chemo, along with lipid mediators within the mitochondria of the heart. To date, lipid mediators within the mitochondria have largely been unexplored.

## 2. Materials and Methods

### 2.1. Experimental Design

This is a secondary analysis from a study testing dietary plant and animal-based omega-3 fatty acids, and sucrose on the brain and liver fatty acids in a mouse model of chemotherapy [41]. This study analyzed the hearts of a subpopulation of 36 ovariectomized female C57BL/6J mice (Charles River Laboratories, Wilmington, DE, USA) to test whether a long-chain omega-3 polyunsaturated fatty acid (LC n-3 PUFA) rich diet (high n-3) can ameliorate the chemotherapy-induced heart damage, and to characterize the fatty acid and lipid mediator differences within the hearts. Mice underwent ovariectomy at 7 weeks of age to simulate post menopause, which is common among females receiving chemotherapy.

Mice were randomized to consume one of two diets: a low n-3 diet (8% by weight fat from soybean oil), or a high n-3 diet (8% by weight fat from soybean oil and MEG-3 powder) (Table S2). Diets were stored under refrigeration in the dark and replaced every 3–4 days to prevent the oxidation of fatty acids. Cumulative food intake was not different between groups.

**Figure 1:**
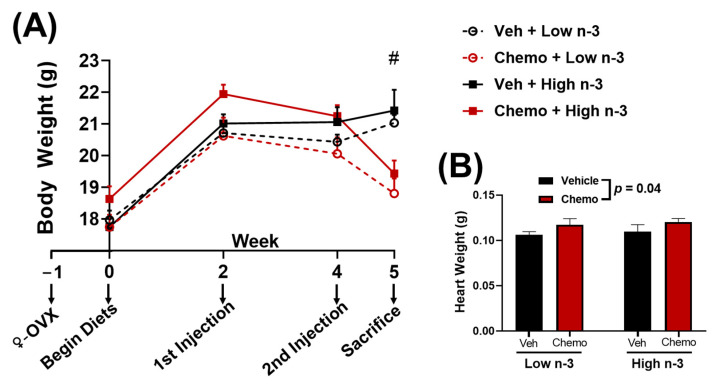
(**A**) Body weight and (**B**) heart weight between groups. Data presented as group means ± SEM (*n* = 9/group). Two-way ANOVA was used to detect differences between groups. #—*p* < 0.05 difference between vehicle and chemo mice. Chemo—9 mg/kg anthracycline + 90 mg/kg cyclophosphamide injections; ♀-OVX—female ovariectomy; Veh—saline vehicle injections; Low n-3—0 g/kg EPA + DHA diet; High n-3—12.2 g/kg EPA + DHA diet (~2% kcal).

Two weeks after diet randomization, mice were further randomized to receive two tail vein chemotherapy injections (9 mg/kg anthracycline, 90 mg/kg cyclophosphamide) or two tail vein saline vehicle injections. Each injection was separated by two weeks. One week after, the final injection mice were anesthetized (Figure 1A). Hearts were perfused with sterile saline before being collected, snap frozen with liquid nitrogen, and stored at −80 °C until further analysis.

Mice were individually housed in a vivarium with room temperature of 22 ± 0.5 °C and on a 14/10-h light/dark cycle, with free access to food and water. All procedures were approved by The Ohio State University Institutional Animal Care and Use Committee (protocol number: 2018A00000053).

### 2.2. Real-Time Quantitative PCR

RNA was isolated from ~50 mg of heart tissue using QIAzol (Quiagen, Hilden, Germanyaccording to the manufacturer’s protocol. RNA concentration was determined (NanoDrop 1000, ThermoFisher Scientific, Waltham, MA, USA) and reverse transcribed to cDNA using a High Capacity cDNA Archive Kit (Applied Biosystems, ThermoFisher Scientific, Waltham, MA, USA). The cDNA was amplified by real-time quantitative PCR with TaqMan Gene Expression Assays using pre-designed and validated primers under universal cycling conditions defined by Applied Biosystems (ThermoFisher Scientific, Waltham, MA, USA). Target gene expression was expressed as 2-ΔΔCT relative to the endogenous control: peptidylprolyl isomerase A (Ppia) [42].

### 2.3. Fatty Acid Analysis

The diet (~0.5 g) and heart (~50 mg) total lipids were extracted with 2:1 (*v/v*) chloroform:methanol using the methodology by Folch et al. [43]. Fatty acid methyl esters were prepared with 5% hydrochloric acid in methanol [44] and analyzed via gas chromatography (GC-2010 or GC-2010 Plus, Shimadzu, Columbia, MD, USA) using a 30 m OmegawaxTM 320 fused silica capillary column (Supelco, Bellefonte, PA, USA), helium as the carrier gas, and flame ionized detector (FID). Oven temperature started at 175 °C and increased at a rate of 3 °C/min until reaching 220 °C. The fatty acid data are reported as a percent of total identified using the area of each fatty acid peak [45].

### 2.4. Mitochondrial Lipid Mediator Analysis

Mitochondria were extracted from flash frozen heart by hand homogenization and centrifugation using the established methodology [46]. Lipids were extracted by modified Smedes extraction [47]. In brief, approximately 30 µg of sample was spiked with 5 µL of 0.1 mg/mL butylated hydroxyl toluene (BHT), ethylenediaminetetraacetic acid (EDTA) in 1:1 methanol:water, and 5 µL of 250 nM deuterated oxylipins and endocannabinoids surrogates (internal standards) in methanol. Next lipids were extracted by the addition of 370 µL of isopropanol, followed by the addition of 470 μL cyclohexane and vertexing. Phases were then separated by the addition of 510 μL of 0.1 M ammonium acetate and centrifugation for 10 min at 12,000 rcf at 0 °C. A total of 400 μL of the top organic layer was then collected and the aqueous phase was reextracted with an additional 520 μL of cyclohexane. The combined organic phase was then dried under vacuum and reconstituted in 40 uL of 1:1 methanol/toluene to form a total lipid extract. Base hydrolysis of complex lipids: To hydrolyze complex lipids, 40 µL of the total lipid extract was incubated with 100 µL 0.5 M sodium methoxide for 1 h at 60 °C, mixed with 100 µL water, and incubated for 1 h at 60 °C. Samples were then diluted with 0.5 mL 0.1% acetic acid/5% methanol and the base was neutralized with 10 µL of 20% glacial acetic acid, and applied onto 10 mg Oasis HLB solid phase extraction column (Waters Corp, Milford, MA, USA) to purify the non-esterified oxylipins, endocannabinoids, and fatty acids. Next, columns were washed with 2 mL of 30% methanol and 0.1% acetic acid in water. Analytes were eluted using 0.5 mL of methanol and 1.5 mL ethyl acetate, dried under vacuum, and reconstituted in 50 µL of 1-cyclohexylureido, 3-dodecanoic acid (CUDA) and 1-phenylureido 3-hexanoic acid (PUHA) at 100 nM in 1:1 methanol:acetonitrile. Prior to targeted liquid chromatography with tandem mass spectrometry (LC-MS/MS), the samples were filtered through a 0.1 µm PVDF spinfilter. Analytical targets were quantified using internal standard methodology against authentic calibration standards detected by electrospray ionization with positive/negative switching and multiple reaction monitoring on an API 6500 QTrap (AB Sciex, Framingham, MA, USA). Briefly, samples were re-randomized for acquisition with blanks, UTAKs, and calibration sets scattered regularly throughout the set. For analysis, 5 μL of the extract was injected and separated using a Shimadzu Nexera X2 UPLC (Shimadzu, Kyoto, Japan) with an Acquity UPLC BEH C18 1.7 μm 2.1 × 100 mm column (Waters, Milford, MA, USA). The mobile phases used were: aqueous0.1% acetic acid in water; polar—9:1 acetonitrile:isopropanol. Samples were held at 10 °C. Separated residues were detected by positive/negative mode switching, with negative mode electrospray ionization for oxylipins and nitro lipids and positive mode electrospray ionization for endocannabinoids and fatty acids using scheduled multiple reaction monitoring on an API 6500 QTRAP (AB Sciex, Framingham, MA, USA). Analytes were quantified using internal standard methods and 6 to 10 point calibration curves (r^2^ ≥ 0.997) with the internal standard used to quantify the extraction surrogate recovery and to establish relative retention times. The calibrants and internal standards were either synthesized [10,11-DHHep, and PUHA] or purchased from Cayman Chemical (Ann Arbor, MI, USA), Medical Isotopes (Pelham, NH, USA), Avanti Polar Lipids Inc. (Alabaster, AL, USA), or Larodan Fine Lipids (Malmö, Sweden). Data were processed with AB Sciex MultiQuant v 3.0.1. Auto-integrations were manually inspected and corrected as necessary. Peaks areas were quantified using response ratios incorporating surrogate peak responses. The LC-MS/MS parameters and standards have previously been described in detail [48].

### 2.5. Statistics

Data were analyzed using a two-way ANOVA with an interaction between the factors: diet (low LC n-3 PUFA vs. high LC n-3 PUFA) and chemo (vehicle injections vs. chemotherapy injections). If the variables contained a significant diet x chemo interaction or significant effects of both the diet and chemo, pairwise comparisons were performed between each group with a Tukey’s post hoc test. Different lowercase letters (a, b, c) were used to signify significant post-hoc differences between groups.

Fold difference was calculated by dividing the lipid mediator concentrations of mice fed one diet with mice fed the comparative diet. Statistical analysis and score plots were made using Stata/IC 15.1 (StataCorp, College Station, TX, USA).

Hierarchical cluster analysis of the mitochondria lipid mediators was performed using the Ward agglomeration and differences in the cluster means were determined using variables as the fixed effect and subject as a random effect. One-way MANOVA was used for the variables with no interaction. Two-way MANOVA with Tukey post testing was used for the variables with significant interaction between diet and chemo. Statistical analysis was performed using JMP (SAS Institute, Cary, NC, USA). *p*-values < 0.05 were reported as significant.

## 3. Results

### 3.1. Chemo Reduced Body Weight and Induced Cardiac Hypertrophy

Compared with the mice given saline, mice given chemo had lower body weights by the end of the study (Figure 1A) as well as greater heart weights (Figure 1B), demonstrating cardiac hypertrophy. The total body and heart weights were not different with LC n-3 PUFA supplementation.

### 3.2. LC n-3 PUFA Supplementation Attenuates Chemo-Induced Markers of Heart Damage and Fibrosis 

**Figure 2:**
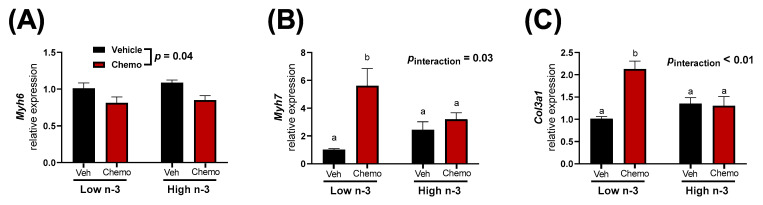
Heart gene expression of (**A**) *Myh6*, (**B**) *Myh7*, and (**C**) *Col3a1*. Data presented as group means ± SEM (*n* = 5/group). A two-way ANOVA with a diet x chemo interaction was used to detect the differences between factors. Pairwise comparisons for variables with significant interactions were performed using a Tukey’s post hoc test. Variables with different annotations were different at *p* < 0.05. Veh—saline vehicle injections; Chemo—9 mg/kg anthracycline + 90 mg/kg cyclophosphamide injections; Low n-3—0 g/kg EPA + DHA diet; High n-3—12.2 g/kg EPA + DHA diet (~2% kcal).

Mice given chemo manifested differential heart damage and fibrosis-related gene expression when compared to the mice given saline vehicle injections. Chemo mice had less *Myh6* expression, while *Myh7* expression was higher, suggesting chemo-induced heart damage (Figure 2A,B). LC n-3 PUFA supplementation did not alter *Myh6* expression in the heart; however, the chemo induced increase of *Myh7* mRNA was attenuated (Figure 2A,B). Additionally, the chemo-induced increase in *Col3a1* expression (a marker of fibrosis) was attenuated with LC n-3 PUFA supplementation (Figure 2C).

### 3.3. Diet and Chemo Alter Cardiac Fatty Acid Composition and Mitochondrial Lipid Metabolites

**Figure 3:**
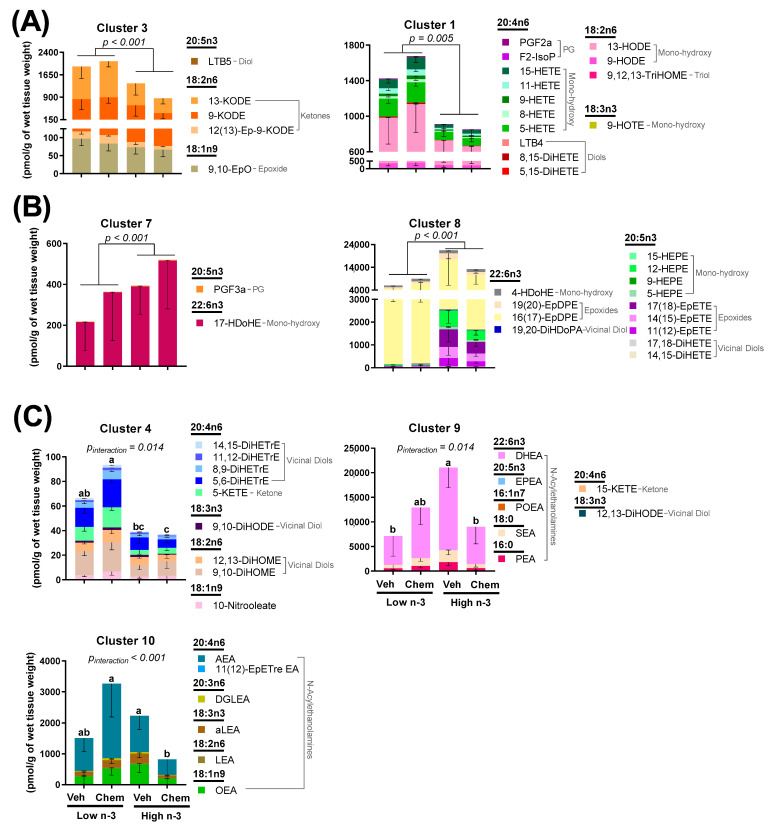
Heart mitochondrial lipid mediator changes between groups using hierarchical cluster analysis. Clusters shown were either (**A**) significantly higher in Low n-3-fed mice; (**B**) significantly higher in High n-3 fed mice; (**C**) or had a significant diet by chemo interaction. Data presented as group means ± SEM (*n* = 4/group). Differences in cluster means were determined using variables as the fixed effect and subject as a random effect. One-way MANOVA was used for the variables with no interaction. Two-way MANOVA with Tukey post testing was used for the variables with significant interaction between diet and chemo (different lowercase letters denote differences in group means). Veh—saline vehicle injections; Chemo—9 mg/kg anthracycline + 90 mg/kg cyclophosphamide injections; Low n-3—0 g/kg EPA + DHA diet; High n-3—12.2 g/kg EPA + DHA diet (~2% kcal). Group means for all measured lipid mediators can be found in Tables S3–S5.

Lipids act as signaling molecules within the heart to control inflammation and cellular energetics. Therefore, the fatty acid composition of the heart (Table 1) as well as lipid mediators in isolated heart mitochondria (Figure 3) were measured to understand how LC n-3 PUFA supplementation and chemo alter lipid pools within the heart. 

Heart fatty acid composition was predominantly altered by diet (Figure S1) though certain fatty acids were altered with chemo (Table 1). Dietary LC n-3 PUFA supplementation led to higher cardiac EPA, DHA, and arachidonic acid. Mice consuming the low LC n-3 PUFA diet had higher oleic, linoleic, and α-linolenic acids, fatty acids that were in higher concentrations in the diet (Table S1). Chemo mice had higher vaccenic (18:1n7), γ-linoleic (18:3n6), and n-3 eicosatetraenoic (20:4n3) acids, while lower n-6 docosapentaenoic acid (22:5n6).

Like fatty acid composition, mitochondrial lipid mediators were predominantly altered by diet (Figure S2). LC n-3 PUFA supplementation resulted in lower concentrations of many arachidonic acid-derived lipid mediators in various lipid classes including a 2.1-fold difference in the prostaglandin (PGF2α), a 2.6-fold difference in mono-hydroxy fatty acids (15-HETE, 11-HETE, 9-HETE, 8-HETE, 5-HETE), a 3.0-fold difference in diols (LTB4, 8,15-DiHETE, 5,15-DiHETE), a 2.0-fold difference in epoxides [14(15)-EpETrE, 11(12)-EpETrE, 8(9)-EpETrE], a 2.4-fold difference in the vicinal diol (14,15-DiHETrE), and a 3.1-fold difference in the ketone (5-KETE). LC n-3 PUFA supplementation resulted in higher concentrations of many EPA-derived lipid mediators in several lipid classes including a 12.4-fold difference in all detected EPA-derived mono-hydroxy fatty acids (15-HEPE, 12-HEPE, 9-HEPE, 5-HEPE), a 15.3-fold difference in all detected epoxides [17(18)-EpETE, 14(15)-EpETE, 11(12)-EpETE], and a 7.2-fold difference in all of the detected vicinal diols (17,18-DiHETE, 14,15-DiHETE) (Tables S3–S5). LTB5 was the only detected EPA-derived lipid mediator that was significantly lower (by 1.3-fold) in mice fed the high n-3 diet (Table S3). Mice given chemo had higher concentrations of two arachidonic acid-derived lipid mediators 5-KETE (1.4-fold difference) and 14,15-DiHETrE (1.4-fold difference), while significant interactions between diet and chemo were common amongst vicinal diols and N-acylethanolamines.

To visualize these broad changes, lipid mediators were aggregated into metabolites showing correlated behavior by hierarchical cluster analysis. Seven of the 10 identified lipid mediator clusters showed significant differences between diet and chemo, as shown in Figure 3. Clusters 1 and 3, which were comprised of linoleic, α-linolenic, and arachidonic acid-derived prostaglandins, mono-hydroxy fatty acids, diols, and ketones were lowered by LC n-3 PUFA supplementation (Figure 3A). Clusters 7 and 8, which were comprised of EPA and DHA-derived mono-hydroxy fatty acids, epoxides, and vicinal diols were increased by LC n-3 PUFA supplementation (Figure 3B). Clusters 4, 9 and 10 (Figure 3C) had significant interactions between diet and chemo, which tended to follow a similar pattern: chemo led to higher concentrations of these lipid mediators in mice consuming the low n-3 diet, while chemo led to lower concentrations of these lipid mediators in mice consuming the high n-3 diet. Of interest, Cluster 4 showed a chemo-mediated enhancement in primarily vicinal diols, metabolites of epoxides generated from sEH, which are lessened by LC n-3 PUFA diet supplementation.

**Figure 4:**
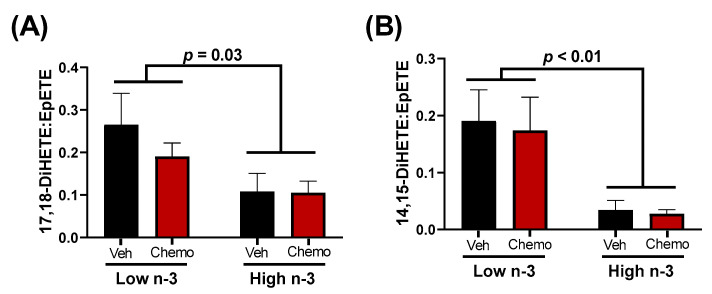
The ratios of the EPA-derived 17,18 (**A**) or 14,15 (**B**) vicinal diol to their corresponding epoxide. Data presented as group means ± SEM (*n* = 4/group). A two-way ANOVA with an interaction was used to detect the differences between groups. Veh—saline vehicle injections; Chemo—9 mg/kg anthracycline + 90 mg/kg cyclophosphamide injections; Low n-3—0 g/kg EPA + DHA diet; High n-3—12.2 g/kg EPA + DHA diet (~2% kcal).

The metabolism between epoxy fatty acids and their corresponding vicinal diol have proven important in cardiovascular pathophysiology [34]. LC n-3 PUFA supplementation led to higher EPA-derived epoxides (15.3-fold difference) and EPA-derived vicinal diols (7.2-fold difference) (Figure 4). It has been suggested that vicinal diols may inhibit beneficial signaling mechanisms of epoxides [32,49]; therefore, the ratio of each vicinal diol to its corresponding parent epoxide was calculated. LC n-3 PUFA supplementation significantly decreased the EPA-derived 17,18-DiHETE:EpETE and 14,15-DiHETE:EpETE ratios, but did not alter any other detectable vicinal diol to epoxide ratio (Figure S3). 

### 3.4. Mitochondrial-Specific Gene Expression Differences

**Figure 5:**
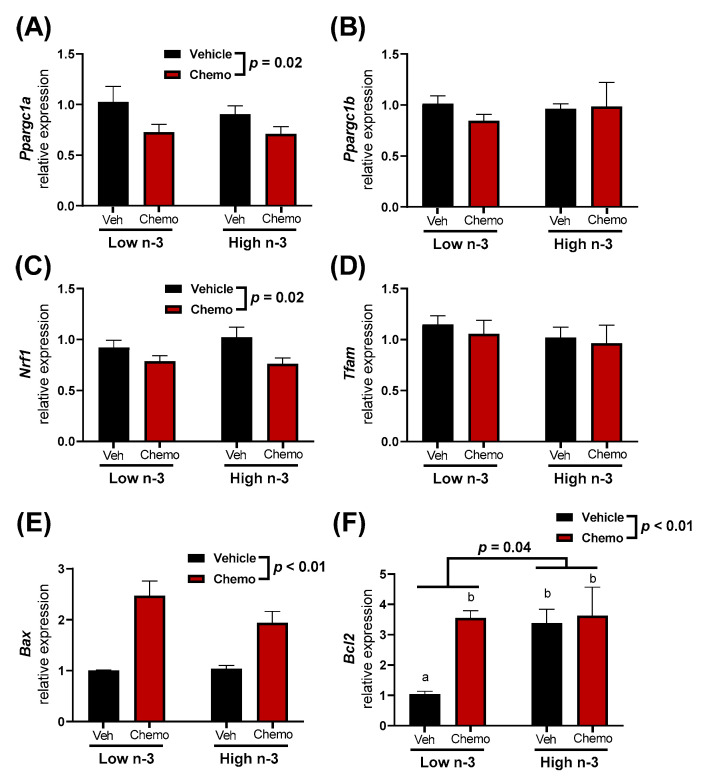
The heart mitochondrial-related gene expression of (**A**) *Ppargc1a*, (**B**) *Ppargc1b*, (**C**) *Nrf1*, (**D**) *Tfam*, (**E**) *Bax*, and (**F**) *Bcl2*. Data presented as group means ± SEM (*n* = 5/group). A two-way ANOVA with a diet x chemo interaction was used to detect the differences between groups while pairwise comparisons were performed using a Tukey’s post hoc test. Group means with different lowercase letters were different at *p* < 0.05. Veh—saline vehicle injections; Chemo—9 mg/kg anthracycline + 90 mg/kg cyclophosphamide injections; Low n-3—0 g/kg EPA + DHA diet; High n-3—12.2 g/kg EPA + DHA diet (~2% kcal).

Mitochondria are central to chemo-induced heart damage. Therefore, several mitochondrial-specific genes in the heart were measured to observe how diet and chemo alter expression (Figure 5). *Ppargc1a* and *Nrf1*, genes important in mitochondrial biogenesis, had lower expression in the hearts of chemo mice, with no differences observed between diets. Mitochondrial apoptotic promoter *Bax*, and the apoptotic inhibitor *Bcl2* had higher expression in the hearts of the chemo mice. *Bcl2* expression was significantly increased with LC n-3 PUFA supplementation, even in the vehicle mice.

## 4. Discussion

Mice were randomized to diets low or high in LC n-3 PUFA (low n-3 and high n-3, respectively) and either chemo or vehicle injections to test whether LC n-3 PUFA supplementation could attenuate chemo-induced markers of cardiotoxicity. Chemo induced body weight loss and increased heart weight (Figure 1B), providing evidence of cardiotoxicity. Gene expression corroborated these findings by revealing chemo-induced myosin heavy chain (MHC) switching from the mature α-isoform to the neonatal β-isoform, characteristic of heart failure (Figure 2A,B) [50]. Additionally, the gene encoding for collagen III was upregulated with chemo, suggesting fibrosis, a structural change in cardiac muscle that contributes to heart failure (Figure 2C) [51]. However, dietary LC n-3 PUFA supplementation attenuated higher *Myh7* and *Col3a1* expression. These molecular markers suggest that LC n-3 PUFA supplementation may reduce certain damaging effects from chemo, thereby preventing fibrosis. Notably, the gene expression of *Myh7* and *Col3a1* appeared to move in a similar fashion to the mitochondrial lipid mediators in Cluster 4, which consisted mostly of vicinal diols (Figure 3C). However, because gene expression and lipid analyses were measured in separate subsets of mice, correlations between the two subsets could not be calculated.

Chemo induces cardiotoxicity in part through mitochondrial dysfunction, lipid peroxidation, and reactive oxygen species formation [10,11]. The cardioprotective properties of LC n-3 PUFA supplementation may be due to lipid composition and lipid mediator changes within the heart [52,53]. Although mitochondria contribute to and metabolize lipid mediators, lipid mediators within mitochondria are largely unexplored [25,26,27]. Therefore, in the present study, cardiac lipid composition, mitochondrial lipid mediators, and their response to LC n-3 PUFA supplementation and chemo were characterized.

Although chemo is known to induce mitochondrial reactive oxygen species and lipid peroxidation, diet contributed to more variability in the heart fatty acid composition and mitochondrial lipid mediators (Figures S1 and S2). Vaccenic (18:1n7) and n-3 eicosatetraenoic acid (20:4n3) increased in the mice given chemo (Table 1). Interestingly, both fatty acids can be generated by the elongase enzyme, ELOVL5. The elevation of vaccenic acid was corroborated by a study in breast cancer patients undergoing chemo, where erythrocyte vaccenic acid was increased after chemo treatment [54]. Additionally, plasma and erythrocyte vaccenic acid was significantly higher in patients with heart failure and is associated with increased mortality and sudden cardiac arrest [55,56]. Further research is needed to explore the mechanism of increased vaccenic acid in response to chemo and whether vaccenic acid plays an active or passive role in disease progression.

LC n-3 PUFA supplementation led to higher arachidonic acid concentrations in the heart (Table 1), but lower amounts of most arachidonic acid-derived mitochondrial lipid mediators (Tables S3–S5) including many that are involved in immune cell activation and chemotaxis (PGF2α, 5-HETE, 20-HETE, LTB4, 5-KETE) [21]. Many of the EPA LOX products (15-HEPE, 12-HEPE, 5-HEPE) were increased with LC n-3 PUFA supplementation. While LC n-3 PUFA supplementation resulted in lower pro-inflammatory arachidonic acid metabolites PGF2α and LTB4, their EPA-derived counterparts PGF3α and LTB5 were either similar or reduced, respectively. This suggests that LC n-3 PUFA supplementation results in fewer pro-inflammatory lipid mediators in heart mitochondria, regardless of their respective parent fatty acids.

Considerable differences in mitochondrial N-acylethanolamines between the chemo and diet groups were identified (Table S5). In the heart, N-acylethanolamines act through various receptors including cannabinoid receptors, peroxisome proliferator-activated receptors, transient receptor potential channels, and G-protein coupled receptors to control vasodilation, immunity, and fibrosis [57]. Little is known regarding the role of N-acylethanolamines within mitochondria. In the current study, similar responses between detected N-acylethanolamines were discovered, regardless of their incorporated fatty acid. Most reported N-acylethanolamines were categorized into Cluster 10 [OEA, LEA, aLEA, AEA, and 11(12)-EpETre EA] and Cluster 9 (PEA, POEA, SEA, EPEA, DHEA) (Figure 3C). Cluster 10 had significantly less mitochondrial lipid mediators in chemo mice fed a high n-3 diet compared with chemo mice fed the low n-3 diet, while Cluster 9 had significantly more mitochondrial lipid mediators in vehicle mice fed the high n-3 diet compared to the vehicle mice fed the low n-3 diet. The alterations in the N-acylethanolamine lipid class suggest the possibility that there is a differential response to chemo-mediated inflammation with LC n-3 PUFA supplementation. More research is needed to better understand the role of N-acylethanolamines with respect to mitochondrial health and cardiac muscle response.

Differences in epoxides and their vicinal diol metabolites between diets present a possible mechanistic link connecting LC n-3 PUFA supplementation to the cardioprotective effects observed herein. Epoxides were demonstrated to be cardioprotective in various models of cardiac dysfunction [30,31,32], while the soluble epoxide hydrolase (sEH)-derived metabolites (vicinal diols) may negate the beneficial effects of epoxides [32,49]. Epoxides have been shown to inhibit cardiac fibroblast proliferation and activation, reduce apoptosis, and preserve mitochondrial function [24,31,32]. In fact, cardiac CYP2J2 overexpression (the enzyme responsible for producing epoxides) was sufficient to improve cardiac function, reduce apoptosis, and preserve mitochondrial function in hearts damaged by anthracyclines [32]. Despite these findings, CYP2J2 overexpression did not attenuate the anthracycline-induced declines in body weight or increases in heart weights, results that corroborate the observations in this study. In the current study, LC n-3 PUFA supplementation led to higher mitochondrial epoxides (Figure 3B, Cluster 8), and lower vicinal diol to epoxide ratios of two EPA-derived oxylipins (Figure 4). Additionally, many vicinal diols were compartmentalized into Cluster 4, which shows a chemo-mediated enhancement in lipid mediators lessened by n-3 diet supplementation (Figure 3). The lower proportionality of vicinal diols to epoxides could be a contributor to the LC n-3 PUFA-mediated cardio-protection observed.

Cardiac mitochondrial-related gene expression was measured to understand how diet and chemo may alter the mitochondrial dynamics (Figure 5). Genes involved in mitochondrial biogenesis (*Ppargc1α* and *Nrf1*) had lower expression in chemo mice, suggesting that chemo impaired mitochondrial biogenesis. Additionally, chemo mice had higher pro-apoptotic *Bax* expression. This suggests a chemo-driven increase in mitochondrial-pathways related to apoptosis within the heart. Interestingly, anti-apoptotic *Bcl2* expression was higher in the LC n-3 PUFA supplemented vehicle mice compared to the vehicle mice fed the low n-3 diet (Figure 5F). Thus, LC n-3 PUFA supplementation may lead to cardiac mitochondria being less prone to apoptosis. Future studies should investigate the possible relationships between these findings and mitochondrial lipid mediators, which increase with LC n-3 PUFA supplementation such as the epoxides identified in Cluster 8 (Figure 3B).

LC n-3 PUFA supplementation attenuated certain chemo-induced markers of heart damage and significantly altered the cardiac fatty acid composition and mitochondrial lipid mediators. Though this study is unique in its approach to measure the lipid mediators specific to isolated mitochondria, there are some limitations that may impact the interpretations. Because of the extraction method of the mitochondria, there may be peroxisomal contamination that was analyzed and presented as the mitochondrial lipidome. Second, it is not possible to attribute any of the observed heart alterations to specific fatty acids or mitochondrial lipid mediators. Finally, the experiment was performed in otherwise healthy mice to isolate alterations specific to chemotherapy. Therefore, the results of this study may change if using a mouse model of cancer. Future research is needed to explore the role of mitochondrial lipid mediators regarding heart function and determine whether LC n-3 PUFA supplementation can ameliorate chemo-induced heart dysfunction by directly measuring the heart function, fibrosis, and mitochondrial dysfunction. 

## Figures and Tables

**Table 1:** Heart fatty acid composition (reported as a percent of total fatty acid detected). Data presented as group means ± SEM (*n* = 4/group). A two-way ANOVA with a diet x chemo interaction was used to detect differences between factors while pairwise comparisons were performed using a Tukey’s post hoc test. *p* < 0.05 are in bold and group means with different letters (a, b, c) are different at *p* < 0.05. Vehicle—saline vehicle injections; Chemo—9 mg/kg anthracycline + 90 mg/kg cyclophosphamide injections; Low n-3—0 g/kg EPA + DHA diet; High n-3—12.2 g/kg EPA + DHA diet (~2% kcal).

	Low n-3		High n-3		Diet X Chemo	Diet	Chemo
	Vehicle	Chemo	Vehicle	Chemo			
10:0	0.12 ± 0.03	0.09 ± 0.01	0.13 ± 0.01	0.14 ± 0.01	0.29	0.11	0.60
12:0	0.09 ± 0.02	0.07 ± 0.01	0.11 ± 0.01	0.11 ± 0.01	0.37	**0.05**	0.34
14:0	0.33 ± 0.06	0.30 ± 0.10	0.40 ± 0.08	0.33 ± 0.03	0.82	0.49	0.47
16:0	13.3 ± 0.3	13.2 ± 0.6	14.4 ± 0.3	14.1 ± 0.2	0.77	**0.03**	0.63
18:0	17.5 ± 0.3	18.3 ± 0.5	18.5 ± 0.4	18.9 ± 0.2	0.59	**0.04**	0.12
20:0	0.39 ± 0.02	0.44 ± 0.01	0.45 ± 0.02	0.45 ± 0.01	0.14	**0.03**	0.09
22:0	0.36 ± 0.01	0.37 ± 0.01	0.32 ± 0.01	0.30 ± 0.01	0.25	**<0.01**	0.37
24:0	0.13 ± 0.01	0.13 ± 0.01	0.13 ± 0.02	0.11 ± 0.01	0.58	0.77	0.61
16:1n7	0.76 ± 0.20	0.53 ± 0.21	0.76 ± 0.11	0.63 ± 0.06	0.74	0.75	0.28
18:1n7	2.0 ± 0.1 ^b^	2.2 ± 0.0 ^ab^	2.2 ± 0.0 ^ab^	2.3 ± 0.0 ^a^	0.98	**<0.01**	**0.05**
18:1n9	8.3 ± 0.8	7.9 ± 1.2	5.2 ± 0.4	5.0 ± 0.4	0.87	**<0.01**	0.69
20:1n9	0.24 ± 0.02	0.25 ± 0.03	0.29 ± 0.03	0.25 ± 0.02	0.32	0.32	0.62
18:2n6	21.8 ± 0.6 ^a^	20.5 ± 0.2 ^a^	10.8 ± 0.3 ^b^	11.8 ± 0.2 ^b^	**0.01**	-	-
18:3n6	0.10 ± 0.01	0.13 ± 0.00	0.19 ± 0.01	0.20 ± 0.01	0.24	**<0.01**	**0.04**
20:2n6	0.33 ± 0.01 ^b^	0.44 ± 0.01 ^a^	0.27 ± 0.01 ^cd^	0.30 ± 0.01 ^bc^	**<0.01**	-	-
20:3n6	0.59 ± 0.04	0.57 ± 0.01	0.32 ± 0.00	0.33 ± 0.02	0.48	**<0.01**	0.84
20:4n6	0.01 ± 0.00	0.00 ± 0.00	0.10 ± 0.02	0.10 ± 0.01	0.85	**<0.01**	0.97
22:4n6	0.52 ± 0.01	0.52 ± 0.02	0.08 ± 0.00	0.06 ± 0.01	0.56	**<0.01**	0.68
22:5n6	1.01 ± 0.13 ^a^	0.81 ± 0.07 ^ab^	0.56 ± 0.01 ^bc^	0.42 ± 0.01 ^c^	0.76	**<0.01**	**0.04**
18:3n3	0.37 ± 0.02	0.32 ± 0.06	0.17 ± 0.02	0.18 ± 0.02	0.42	**<0.01**	0.61
18:4n3	0.11 ± 0.01	0.10 ± 0.00	0.13 ± 0.01	0.13 ± 0.01	0.53	**0.01**	0.61
20:4n3	8.3 ± 0.3	8.8 ± 0.3	2.7 ± 0.0	3.8 ± 0.1	0.18	**<0.01**	**<0.01**
20:5n3	0.06 ± 0.01	0.07 ± 0.01	1.21 ± 0.06	1.30 ± 0.06	0.37	**<0.01**	0.28
22:5n3	0.9 ± 0.1 ^b^	1.1 ± 0.1 ^b^	2.0 ± 0.1 ^a^	1.8 ± 0.0 ^a^	**0.03**	-	-
22:6n3	22.3 ± 0.6	22.8 ± 1.3	38.6 ± 0.7	36.9 ± 0.6	0.22	**<0.01**	0.53

## Data Availability

The data presented in this study are available on requestion from the corresponding author.

## Supplementary Materials

The following supporting information can be downloaded at: https://www.mdpi.com/article/10.3390/metabo12090782/s1, Figure S1: Score plot from the principal component analysis of the heart fatty acid composition; Figure S2: Score plot from the principal component analysis of heart mitochondrial lipid mediators; Figure S3: Ratios of vicinal diol to their parent epoxide; Table S1: Composition of experimental diets; Table S2: Fatty acid composition of the experimental diets; Table S3: Heart mitochondrial lipid mediators (part 1 of 3); Table S4: Heart mitochondrial lipid mediators (part 2 of 3); Table S5: Heart mitochondrial lipid mediators (part 3 of 3).
