# Supplementary material for: Potential Cardioprotective Effects and Lipid Mediator Differences in Long-Chain Omega-3 Polyunsaturated Fatty Acid Supplemented Mice Given Chemotherapy
Source: Metabolites. 2022 Aug 24;12(9):782. doi: 10.3390/metabo12090782 (PMC9505633; doi:10.3390/metabo12090782)
Supplement: Supplementary file 1 [file metabolites-12-00782-s001.zip › metabolites-1843500-supplementary.pdf]

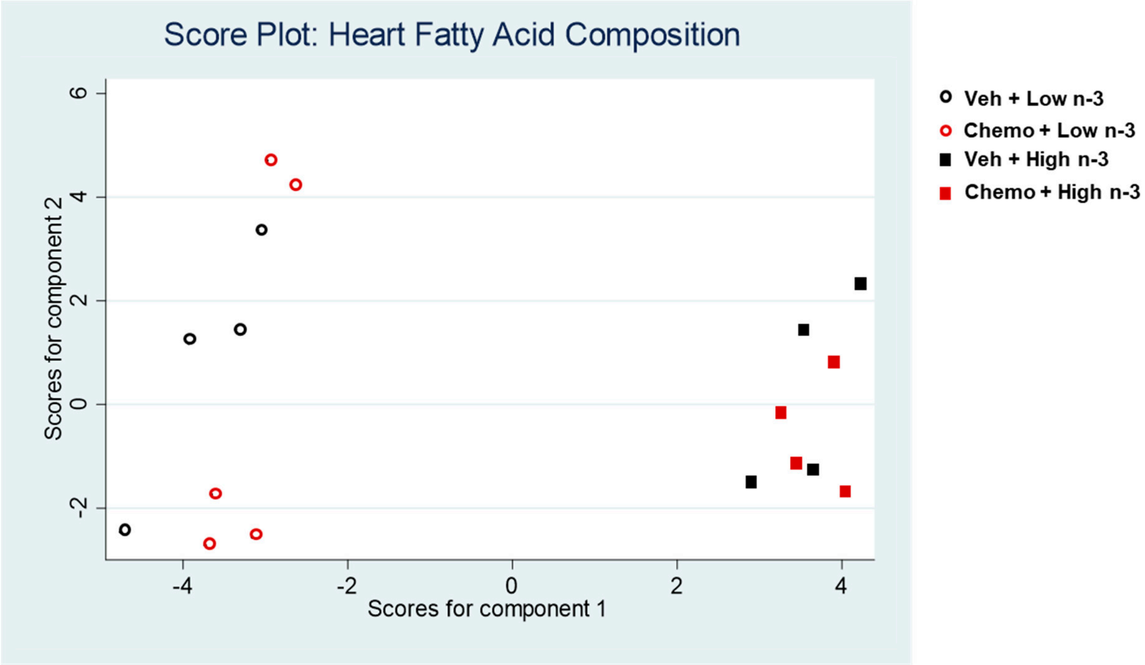

Figure S1: Scores plot from the principal component analysis of the heart fatty acid composition. Graph was made using Stata/IC 15.1 (StataCorp College Station, TX). Veh – saline vehicle injections; Chemo – 9 mg/kg anthracycline + 90 mg/kg cyclophosphamide injections; Low n-3 – 0 g/kg EPA+DHA diet; High n-3 – 12.2 g/kg EPA+DHA diet (~2% kcal).

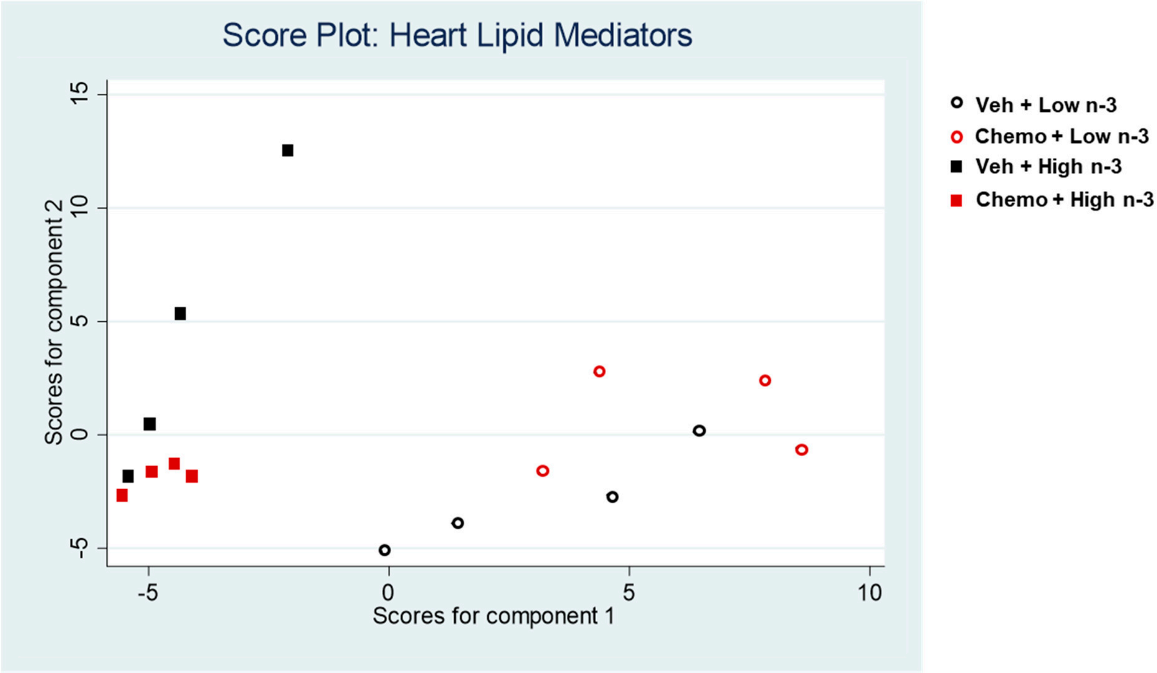

Figure S2: Score plot from the principal component analysis of the heart mitochondrial lipid mediators. Graph was made using Stata/IC 15.1 (StataCorp College Station, TX). Veh – saline vehicle injections; Chemo – 9 mg/kg anthracycline + 90 mg/kg cyclophosphamide injections; Low n-3 – 0 g/kg EPA+DHA diet; High n-3 – 12.2 g/kg EPA+DHA diet (~2% kcal).

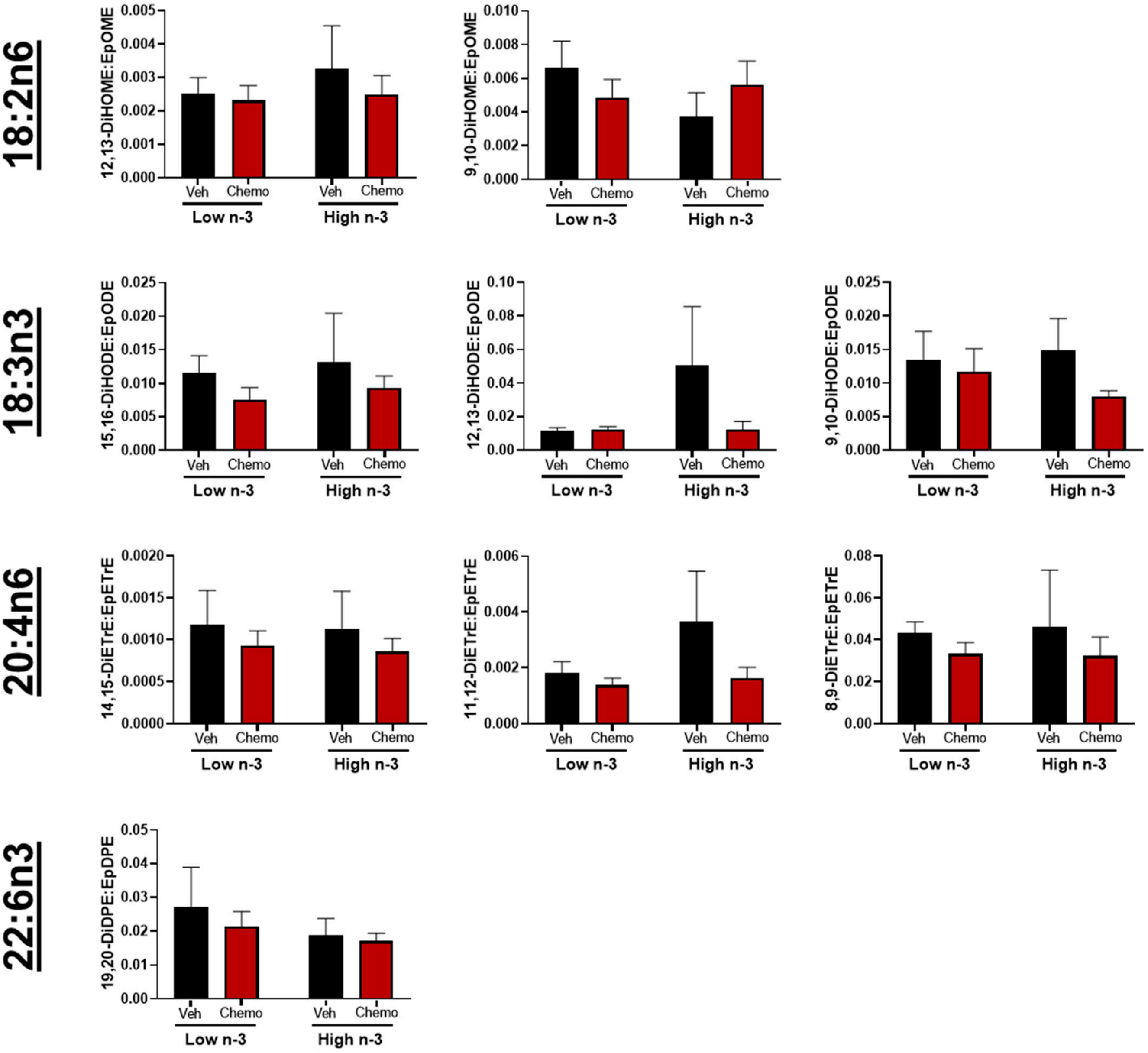

Figure S3. Ratios of vicinal diol to their parent epoxide. Data presented as group means ± SEM (n = 4/group). A two-way ANOVA with an interaction was used to detect the differences between groups. Veh – saline vehicle injections; Chemo – 9 mg/kg anthracycline + 90 mg/kg cyclophosphamide injections; Low n-3 – 0 g/kg EPA+DHA diet; High n-3 – 12.2 g/kg EPA+DHA diet (~2% kcal).

Table S1. Composition of experimental diets (prepared by Research Diets Inc., RDI)

**RDI Product #**	**D13091002B**	**D15082608**
	Low n-3 Diet	High n-3 Diet
	**Diet 4**	**Diet 2**
**Ingredient**	**gm**	**Gm**
Casein	232.5	205
DL-Methionine	3	3
Corn Starch	450	450
Sucrose	100	100
Maltodextrin 10	100	100
Cellulose, BW200	50	50
Soybean Oil	90	41
MEG-3, 30% Powder	0	81
Corn Oil	0	0
Mineral Mix S10001	35	35
Vitamin Mix V10001	10	10
Choline Bitartrate	2	2
FD&C Yellow Dye #5	0.025	0
FD&C Red Dye #40	0	0.05
FD&C Blue Dye #1	0.025	0
**Total**	**1072.55**	**1072.05**
Protein (gm)	205.3	205.2
Carbohydrate (gm)	650	650
Fat (gm)	90	89.8
Fiber (gm)	50	50
Protein (kcal)	821.1	820.6
Carbohydrate (kcal)	2600	2600
Fat (kcal)	810	808.2
Total kcal	4231.1	4228.8
Protein (gm%)	19	19
Carbohydrate (gm%)	61	61
Fat (gm%)	8	8
Protein(kcal%)	19	19
Carbohydrate(kcal%)	61	61
Fat (kcal%)	19	19

Table S2. Fatty acid composition of the experimental diets. Values are averages of triplicate analysis by gas chromatography, ND - Not detectable.

	**Low n-3 Diet**		**High n-3 Diet**	
	Mean %	*SD*	*Mean %*	*SD*
**C14:0**	**0.4285**	**0.0643**	**3.9649**	**0.1351**
**C16:0**	**11.0826**	**0.1633**	**15.8085**	**0.1126**
**C16:1n7**	**0.1121**	**0.0067**	**4.6269**	**0.1143**
**C16:2n4**	**ND**	**ND**	**0.6594**	**0.0220**
**C16:3n4**	**0.0388**	**0.0000**	**0.7631**	**0.0158**
**C18:0**	**4.0678**	**0.1251**	**3.9742**	**0.0336**
**C18:1n9**	**21.2491**	**0.3757**	**15.1007**	**0.1886**
**C18:1n7**	**1.4156**	**0.0212**	**2.1620**	**0.0064**
**C18:2n6**	**54.6681**	**0.3481**	**27.4554**	**0.5124**
**C18:3n6**	**ND**	**ND**	**0.1747**	**0.0035**
**C18:3n3**	**6.3831**	**0.1438**	**3.6822**	**0.0622**
**C18:4n3**	**ND**	**ND**	**2.0039**	**0.0317**
**C20:0**	**0.2941**	**0.0210**	**0.4932**	**0.0033**
**C20:1n9**	**0.2860**	**0.0373**	**0.9943**	**0.0053**
**C20:2n6**	**ND**	**ND**	**0.1234**	**0.0016**
**C20:3n6**	**ND**	**ND**	**0.0987**	**0.0045**
**C20:4n6**	**ND**	**ND**	**0.4923**	**0.0024**
**C20:4n3**	**ND**	**ND**	**0.5551**	**0.0113**
**C20:5n3**	**ND**	**ND**	**9.0097**	**0.1618**
**C22:4n6**	**ND**	**ND**	**ND**	**ND**
**C22:5n6**	**ND**	**ND**	**0.1502**	**0.0035**
**C22:5n3**	**ND**	**ND**	**0.8362**	**0.0125**
**C22:6n3**	**ND**	**ND**	**6.8710**	**0.1857**
**Total n-3**	**6.3831**	**0.1438**	**11.9445**	**0.1466**
**Total n-6**	**54.6681**	**0.3481**	**28.3200**	**0.5092**
**n-3/n-6**	**0.1168**	**0.0021**	**0.4219**	**0.0127**
**n-6/n-3**	**8.5668**	**0.1575**	**2.3715**	**0.0717**
**Sat FA**	**15.8731**	**0.2113**	**24.2408**	**0.2170**
**MUFA**	**23.0628**	**0.4124**	**22.8839**	**0.0816**
**PUFA**	**61.0512**	**0.4664**	**50.1083**	**0.1800**

Table S3. Heart mitochondrial lipid mediators (part 1 of 3). Unless otherwise noted units are in pmol/g. Low extraction efficiency or uncalibrated analytes are reported as relative abundance (%) across the experiment. Data is presented as group means ± SEM (*n* = 4/group). A two-way ANOVA with a diet x chemo interaction was used to detect the differences between factors. P<0.05 are bolded. PGs-prostaglandins. Vehicle – saline vehicle injections; Chemo – 9 mg/kg anthracycline + 90 mg/kg cyclophosphamide injections; Low n-3 – 0 g/kg EPA+DHA diet; High n-3 – 12.2 g/kg EPA+DHA diet (~2% kcal).

			Low n-3		High n-3		Diet X Chemo	Diet	Chemo
			Vehicle	Chemo	Vehicle	Chemo			
PGs	20:4n6	PGF$_{2\alpha}$ (%)	6.31 ± 1.07	7.36 ± 1.85	3.35 ± 1.21	3.07 ± 0.81	0.62	**0.02**	0.77
		F2 Isoprostane (%)	4.55 ± 0.71	4.99 ± 0.83	4.40 ± 0.77	4.18 ± 0.93	0.69	0.57	0.90
	20:5n3	PGF$_{3\alpha}$ (%)	2.87 ± 0.32	3.42 ± 0.58	4.05 ± 0.53	4.33 ± 0.78	0.82	0.10	0.49
Mono-Hydroxy Fatty Acids	18:2n6	13-HODE	632 ± 125	730 ± 159	491 ± 117	438 ± 90	0.56	0.11	0.86
		9-HODE	345 ± 67	400 ± 99	229 ± 58	219 ± 48	0.66	0.06	0.75
	18:3n3	13-HOTE	12.4 ± 7.48	6.18 ± 1.19	4.70 ± 1.10	3.43 ± 0.66	0.54	0.20	0.35
		9-HOTE	3.58 ± 0.56	3.37 ± 0.85	3.09 ± 0.59	2.23 ± 0.33	0.60	0.20	0.40
	20:4n6	15-HETE	100 ± 23.1	136 ± 34.7	36.4 ± 5.8	41.5 ± 7.9	0.49	**<0.01**	0.36
		12-HETE	883 ± 730	315 ± 123	139 ± 54	87 ± 30	0.50	0.22	0.42
		11-HETE	60.7 ± 15.4	69.3 ± 12.9	17.5 ± 2.4	21.9 ± 4.0	0.84	**<0.01**	0.54
		9-HETE	28.9 ± 5.1	41.1 ± 8.1	14.5 ± 2.1	16.7 ± 2.6	0.34	**<0.01**	0.18
		8-HETE	26.9 ± 5.1	35.3 ± 7.4	12.7 ± 1.7	13.5 ± 2.0	0.43	**<0.01**	0.34
		5-HETE	201 ± 27	227 ± 23	91 ± 11	84 ± 7	0.40	**<0.01**	0.64
	20:5n3	15-HEPE	1.8 ± 0.2	1.9 ± 0.3	14.2 ± 2.8	11.1 ± 1.5	0.31	**<0.01**	0.37
		12-HEPE	51 ± 27	40 ± 9	695 ± 321	394 ± 113	0.41	**0.01**	0.38
		9-HEPE	0.7 ± 0.2	1.0 ± 0.1	12.7 ± 1.9	10.5 ± 1.2	0.29	**<0.01**	0.42
		5-HEPE	6.8 ± 1.1	6.4 ± 0.6	117 ± 16	95.5 ± 7.6	0.24	**<0.01**	0.22
	22:6n3	17-HdoHE	214 ± 48	361 ± 95	389 ± 62	515 ± 97	0.90	0.058	0.11
		14-HdoHE	654 ± 555	298 ± 93	763 ± 273	501 ± 104	0.88	0.63	0.35
		4-HdoHE	792 ± 72	1125 ± 166	1340 ± 121	1256 ± 176	0.16	**0.03**	0.39
Diols	20:4n6	LTB4	1.35 ± 0.24	1.33 ± 0.31	0.86 ± 0.06	0.62 ± 0.06	0.59	**0.01**	0.52
		6-trans-LTB4	2.36 ± 1.15	1.62 ± 0.33	0.95 ± 0.14	0.52 ± 0.07	0.79	0.06	0.35
		8,15-DiHETE	9.51 ± 3.14	14.4 ± 3.94	3.63 ± 0.52	3.98 ± 0.54	0.39	**0.01**	0.33
		5,15-DiHETE	1.34 ± 0.49	1.85 ± 0.65	0.47 ± 0.11	0.46 ± 0.10	0.55	**0.02**	0.56
	20:5n3	LTB5	0.22 ± 0.04	0.20 ± 0.05	0.11 ± 0.02	0.09 ± 0.01	0.98	**0.01**	0.53
Triol	18:2n6	9,12,13-TriHOME (%)	2.28 ± 0.40	2.89 ± 0.51	3.59 ± 0.77	2.38 ± 0.50	0.13	0.49	0.61

Table S4. Heart mitochondrial lipid mediators (part 2 of 3). Unless otherwise noted units are in pmol/g. Data is presented as group means ± SEM (*n* = 4/group). A two-way ANOVA with a diet x chemo interaction was used to detect the differences between factors. p<0.05 are bolded. Vehicle – saline vehicle injections; Chemo – 9 mg/kg anthracycline + 90 mg/kg cyclophosphamide injections; Low n-3 – 0 g/kg EPA+DHA diet; High n-3 – 12.2 g/kg EPA+DHA diet (~2% kcal).

			Low n-3		High n-3		Diet X Chemo	Diet	Chemo
			Vehicle	Chemo	Vehicle	Chemo			
Epoxides	18:1n9	9(10)-EpO	96.9 ± 8.3	83.3 ± 9.8	73.4 ± 7.2	66.8 ± 6.1	0.67	**0.03**	0.23
	18:2n6	12(13)-EpOME	3010 ± 534	4535 ± 536	3279 ± 1254	2415 ± 525	0.15	0.26	0.68
		12(13)-Ep-9-KODE	20.1 ± 3.3	23.5 ± 1.0	14.6 ± 2.2	10.0 ± 0.8	0.09	**<0.01**	0.80
		9(10)-EpOME	3453 ± 700	5293 ± 740	4057 ± 1816	2515 ± 611	0.15	0.34	0.89
	18:3n3	15(16)-EpODE	284 ± 93	350 ± 95	383 ± 203	175 ± 16	0.28	0.76	0.57
		12(13)-EpODE	42.3 ± 9.8	58.9 ± 4.0	53.1 ± 20.3	37.5 ± 7.4	0.21	0.67	0.97
		9(10)-EpODE	161 ± 52	208 ± 41	192 ± 94	103 ± 12	0.26	0.54	0.71
	20:4n6	14(15)-EpETrE	1904 ± 605	2923 ± 490	1188 ± 482	1163 ± 309	0.30	**0.03**	0.32
		11(12)-EpETrE	964 ± 249	1628 ± 286	700 ± 300	604 ± 166	0.16	**0.03**	0.29
		11(12)-EpETrE EA	1.99 ± 0.20	4.87 ± 1.03	2.40 ± 0.81	1.22 ± 0.11	**0.01**	-	-
		8(9)-EpETrE	114 ± 20	243 ± 47	107 ± 36	100 ± 29	0.07	**0.05**	0.10
	20:5n3	17(18)-EpETE	14 ± 3	23 ± 4	774 ± 317	512 ± 105	0.44	**<0.01**	0.46
		14(15)-EpETE	25 ± 6	40 ± 14	491 ± 208	340 ± 69	0.47	**0.01**	0.55
		11(12)-EpETE	30 ± 11	43 ± 18	352 ± 154	222 ± 44	0.39	**0.01**	0.48
	22:6n3	19(20)-EpDPE	708 ± 256	843 ± 205	2564 ± 1325	1257 ± 193	0.32	0.13	0.41
		16(17)-EpDPE	4385 ± 1178	6790 ± 1158	14997 ± 6479	9043 ± 2059	0.26	0.09	0.62
Vicinal Diols	18:2n6	12,13-DiHOME	6.91 ± 0.18	10.1 ± 1.70	6.70 ± 0.51	5.27 ± 0.44	**0.03**	-	-
		9,10-DiHOME	19.5 ± 0.8	23.8 ± 3.3	9.3 ± 1.9	11.8 ± 1.0	0.67	**<0.01**	0.12
	18:3n3	15,16-DiHODE	2.71 ± 0.51	2.25 ± 0.24	2.42 ± 0.48	1.56 ± 0.21	0.61	0.23	0.12
		12,13-DiHODE	0.48 ± 0.14	0.74 ± 0.15	1.15 ± 0.20	0.36 ± 0.07	**<0.01**	-	-
		9,10-DiHODE	1.60 ± 0.15	2.15 ± 0.45	1.73 ± 0.34	0.81 ± 0.11	**0.03**	-	-
	20:4n6	14,15-DiHETrE	1.66 ± 0.12	2.48 ± 0.30	0.81 ± 0.10	0.89 ± 0.19	0.09	**<0.01**	**0.04**
		11,12-DiHETrE	1.46 ± 0.09	2.05 ± 0.11	1.36 ± 0.09	0.81 ± 0.07	**<0.01**	-	-
		8,9-DiHETrE	4.68 ± 0.62	7.37 ± 0.45	2.57 ± 0.26	2.54 ± 0.13	**0.01**	-	-
		5,6-DiHETrE	15.6 ± 2.32	22.73 ± 1.52	10.23 ± 1.47	6.79 ± 0.37	**0.01**	-	-
	20:5n3	17,18-DiHETE	3.16 ± 0.43	4.02 ± 0.46	50.3 ± 5.1	45.8 ± 3.03	0.38	**<0.01**	0.55
		14,15-DiHETE	3.77 ± 0.47	4.83 ± 0.88	9.35 ± 0.98	8.00 ± 0.41	0.13	**<0.01**	0.85
	22:6n3	19,20-DiHDoPA	11.9 ± 1.0	15.4 ± 1.2	28.6 ± 2.9	20.4 ± 1.5	**0.01**	-	-

Table S5. Heart mitochondrial lipid mediators (part 3 of 3). Unless otherwise noted units are in pmol/g. Low extraction efficiency or uncalibrated analytes are reported as relative abundance (%) across the experiment. Data is presented as group means ± SEM (*n* = 4/group). A two-way ANOVA with a diet x chemo interaction was used to detect the differences between factors. p<0.05 are bolded. Vehicle – saline vehicle injections; Chemo – 9 mg/kg anthracycline + 90 mg/kg cyclophosphamide injections; Low n-3 – 0 g/kg EPA+DHA diet; High n-3 – 12.2 g/kg EPA+DHA diet (~2% kcal).

			Low n-3		High n-3		Diet X Chemo	Diet	Chemo
			Vehicle	Chemo	Vehicle	Chemo			
Ketones	18:2n6	13-KODE	1088 ± 116	1209 ± 75	737 ± 193	497 ± 79	0.17	**<0.01**	0.64
		9-KODE	716 ± 86	786 ± 76	535 ± 186	291 ± 81	0.20	**0.01**	0.47
		12(13)-Ep-9-KODE	20.1 ± 3.3	23.5 ± 1.0	14.6 ± 2.2	10.0 ± 0.8	0.09	**<0.01**	0.80
	20:4n6	15-KETE	39.0 ± 0.9	53.9 ± 2.5	63.1 ± 10.5	37.2 ± 7.9	**0.01**	-	-
		5-KETE	11.2 ± 1.7	16.3 ± 1.3	4.0 ± 1.0	4.9 ± 0.5	0.11	**<0.01**	**0.03**
N-Acylethanolamines	16:0	PEA	503 ± 18	994 ± 214	1728 ± 385	556 ± 67	**<0.01**	-	-
	18:0	SEA	737 ± 83	1579 ± 338	2465 ± 544	828 ± 126	**<0.01**	-	-
	16:1n7	POEA (%)	2.14 ± 0.35	3.11 ± 0.81	13.16 ± 2.60	3.18 ± 0.65	**<0.01**	-	-
	18:1n9	OEA	264 ± 23	517 ± 113	660 ± 153	199 ± 33	**<0.01**	-	-
	18:2n6	LEA	25.1 ± 1.7	48.1 ± 11.7	43.9 ± 9.6	15.3 ± 2.2	**0.01**	-	-
	18:3n3	aLEA	129 ± 15	227 ± 57	298 ± 71	86 ± 19	**0.01**	-	-
	20:3n6	DGLEA	26.1 ± 1.0	62.6 ± 18.5	54.6 ± 17.5	13.7 ± 1.8	**0.01**	-	-
	20:4n6	AEA	1064 ± 102	2405 ± 616	1166 ± 231	503 ± 71	**0.01**	-	-
		11(12)-EpETrE EA	1.99 ± 0.20	4.87 ± 1.03	2.40 ± 0.81	1.22 ± 0.11	**0.01**	-	-
	20:5n3	EPEA (%)	0.27 ± 0.03	0.64 ± 0.18	18.73 ± 4.60	5.34 ± 1.10	**0.01**	-	-
	22:5n6	DEA	34575 ± 14112	10548 ± 5468	10097 ± 8275	7196 ± 4302	0.26	0.14	0.16
	22:6n3	DHEA	5850 ± 150	10298 ± 1750	16775 ± 2357	7605 ± 1013	**<0.01**	-	-
	18:1n9	10-Nitrooleate	3.71 ± 0.44	6.63 ± 1.12	2.56 ± 0.58	3.26 ± 1.28	0.26	**0.03**	0.08
	18:1n9	NO-Gly	0.09 ± 0.04	0.14 ± 0.04	0.10 ± 0.02	0.12 ± 0.07	0.77	0.88	0.48
